# Community pharmacist attitudes towards collaboration with general practitioners: development and validation of a measure and a model

**DOI:** 10.1186/1472-6963-12-320

**Published:** 2012-09-16

**Authors:** Connie Van, Daniel Costa, Penny Abbott, Bernadette Mitchell, Ines Krass

**Affiliations:** 1 Faculty of Pharmacy, The University of Sydney, Sydney, Australia; 2 School of Psychology, The University of Sydney, Sydney, Australia; 3 School of Medicine, University of Western Sydney, Sydney, Australia

## Abstract

**Background:**

Community Pharmacists and General Practitioners (GPs) are increasingly being encouraged to adopt more collaborative approaches to health care delivery as collaboration in primary care has been shown to be effective in improving patient outcomes. However, little is known about pharmacist attitudes towards collaborating with their GP counterparts and variables that influence this interprofessional collaboration. This study aims to develop and validate 1) an instrument to measure pharmacist attitudes towards collaboration with GPs and 2) a model that illustrates how pharmacist attitudes (and other variables) influence collaborative behaviour with GPs.

**Methods:**

A questionnaire containing the newly developed “Attitudes Towards Collaboration Instrument for Pharmacists” (ATCI-P) and a previously validated behavioural measure “Frequency of Interprofessional Collaboration Instrument for Pharmacists” (FICI-P) was administered to a sample of 1215 Australian pharmacists. The ATCI-P was developed based on existing literature and qualitative interviews with GPs and community pharmacists. Principal Component Analysis was used to assess the structure of the ATCI-P and the Cronbach’s alpha coefficient was used to assess the internal consistency of the instrument. Structural equation modelling was used to determine how pharmacist attitudes (as measured by the ATCI-P) and other variables, influence collaborative behaviour (as measured by the FICI-P).

**Results:**

Four hundred and ninety-two surveys were completed and returned for a response rate of 40%. Principal Component Analysis revealed the ATCI-P consisted of two factors: ‘interactional determinants’ and ‘practitioner determinants’, both with good internal consistency (Cronbach’s alpha = .90 and .93 respectively). The model demonstrated adequate fit (χ^2^/df = 1.89, CFI = .955, RMSEA = .062, 90% CI [.049-.074]) and illustrated that ‘interactional determinants’ was the strongest predictor of collaboration and was in turn influenced by ‘practitioner determinants’. The extent of the pharmacist’s contact with physicians during their pre-registration training was also found to be a significant predictor of collaboration (B = .23, SE = .43, p <.001).

**Conclusions:**

The results of the study provide evidence for the validity of the ATCI-P in measuring pharmacist attitudes towards collaboration with GPs and support a model of collaboration, from the pharmacist’s perspective, in which collaborative behaviour is influenced directly by ‘interactional’ and ‘environmental determinants’, and indirectly by ‘practitioner determinants’.

## Background

Interprofessional collaboration involves individuals from different disciplines working together while contributing to patient care from their own professional perspective [1]. It is now widely recognised that in order to provide adequate care for chronically ill patients, health providers must work together to improve and prevent gaps in service delivery [2]. General practitioners (GPs) and community pharmacists are increasingly being encouraged to adopt more collaborative approaches to health care delivery as collaboration between GPs and pharmacists has been shown to be an effective means of improving patient care by helping patients achieve therapeutic goals [3-5] and enhancing medication management [6-8]. Research to date has tended to focus on the effect of GP-pharmacist collaboration on patient outcomes [9-11]. Limited attention has been paid to GP and pharmacist attitudes towards collaboration. This subject is important as a practitioner’s attitudes towards collaboration may influence the degree to which they collaborate with their counterpart.

### Existing measures of attitudes towards interprofessional collaboration

Currently, only one validated measure of pharmacist-physician collaboration exists. The “Physician-Pharmacist Collaboration Instrument” developed by Zillich et al. [12] for physicians is a 14-item instrument developed using literature from interpersonal, business, and health care relationships and covers seven themes: collaborative care, commitment, dependence, symmetry, bidirectional communication, trust, initiating behaviour, and conflict resolution. The instrument was tested and validated on a sample of US physicians practicing in family medicine, internal medicine and paediatrics. The instrument was later adapted for pharmacists, and tested for sensitivity and criterion validity on a small (n = 25) sample of community pharmacists [13]. More recently, Hojat & Gonnella [14] developed the “Scale of Attitudes Towards Pharmacist-Physician Collaboration.” The instrument has yet to be validated but preliminary psychometric analysis has been carried out on a small (n = 42) sample of retail pharmacists, hospital pharmacists and physicians affiliated with a tertiary institution. The 16-item instrument was designed for administration to pharmacists, physicians and students of pharmacy and medicine. The instrument was based on ‘frequently mentioned components of collaboration in the literature’ and some items were adapted from an existing physician-nurse collaboration instrument.

Although the two instruments discussed above cover many dimensions of collaboration (many of which are relevant to the primary care setting), they were not specifically designed for community pharmacists practicing in primary care and therefore do not address all dimensions of collaboration relevant to this sector. For example the “Physician-Pharmacist Collaboration Instrument” [12] does not address lack of GP recognition of the pharmacist’s role in medication management - a theme that has been frequently reported as a barrier to collaboration between physicians and pharmacists practising in primary care [15-18] and the “Scale of Attitudes Towards Pharmacist-Physician Collaboration” [14] does not cover the dimension of trust. Each instrument also has other limitations worth noting. The “Physician-Pharmacist Collaboration Instrument” [12] was developed and validated using one group of practitioners only i.e. physicians. The instrument must be modified for pharmacists but has only been tested on a small sample for this purpose [13]. On the other hand, the “Scale of Attitudes Towards Pharmacist-Physician Collaboration” [14] was designed to be administered to physicians and pharmacists practicing in various settings, as well as students. As a result the instrument is more general i.e. the items refer to physicians and pharmacists in general terms as the same items need to be applicable to a broad population. Due to this design, the instrument does not tap into important personal aspects of a collaborative relationship such as trust and respect between two practitioners.

### Existing theoretical models for interprofessional collaboration

The drive for collaboration in the provision of healthcare has also led to the development of a number of models that conceptualise collaborative behaviour [19-23]. These models include those that describe factors influencing collaboration as well as those that describe the different stages and characteristics of collaboration mostly in a nursing context. Only a limited number of models have described the dynamics of collaboration between physicians and pharmacists [24,25]. The “Collaborative working relationship model”, proposed by McDonough and Doucette [24], is a general model for physicians and pharmacists. The model describes collaboration as an evolving process consisting of five stages: stage 0 – professional awareness, stage 1 – professional recognition, stage 2 – exploration and trial, stage 3 – professional relationship expansion and stage 4 – commitment to the collaborative working relationship. The model also posits that the drivers of collaboration include participant, context and exchange characteristics. More recently, Bradley et al. [25] developed a “Conceptual model of GP-pharmacist collaboration” specifically for GPs and community pharmacists, consisting of three stages: stage 1 – isolation, stage 2 – communication and stage 3 – collaboration, with key components of collaboration identified as locality, service provision, trust, ‘knowing’ each other, communication, professional roles and respect.

The stages of the “Collaborative working relationship model” was synthesised from existing models of interpersonal relationships, including theories of social exchange, business relationships and collaborative care models primarily relating to nurses and physicians, rather than drawn from physician and pharmacist’s own accounts of their collaborative relationships [24]. The proposed drivers of collaboration however have been tested [26]. In contrast, the “Conceptual model of GP-pharmacist collaboration” was derived from interviews with GPs and community pharmacists in the UK however the model has not yet been tested [25].

### Study aims

The aims of the study were to develop and validate, in the context of primary care in Australia 1) an instrument to measure pharmacist attitudes towards collaborating with GPs and 2) a model that illustrates how pharmacist attitudes (and other variables) influence collaborative behaviour with GPs.

A corresponding instrument that measures GP attitudes towards collaborating with pharmacists will be reported in a separate paper (manuscript under review).

## Methods

Ethics approval was obtained from the University of Sydney Human Ethics Committee prior to commencement of the study.

### Theoretical background

Initially, qualitative interviews with GPs and pharmacists were conducted to explore the nature, extent and determinants of collaborative interactions between pharmacists and GPs. Details of this study have been published in an earlier paper [15]. To summarise, interactions between pharmacists and GPs involved administrative issues associated with the dispensing process, exchange of patient and drug information and discussion of patients’ drug therapy. Factors which appeared to influence collaborative behaviour concurred with previous studies [26-33], and included either ‘interactional’, ‘practitioner’ or ‘environmental determinants’. ‘Interactional determinants’ comprise those components of interpersonal relationships, ‘practitioner determinants’ include elements related to the GP or pharmacist individually and ‘environmental determinants’ describe the conditions under which the practitioner practices.

**Figure 1:**
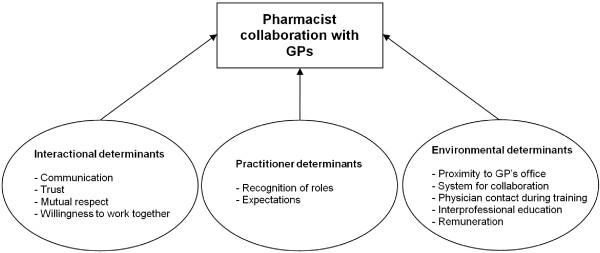
Theoretical model showing factors influencing pharmacist collaboration with GPs.

Based on the qualitative interviews [15] and existing literature on interprofessional collaboration [20-24,34,35], a hypothesised model for collaboration between pharmacists and GPs was developed (Figure 1).

### Development of the attitudes towards collaboration instrument for pharmacists

Generation of the items of the Attitudes Towards Collaboration Instrument for Pharmacists (ATCI-P) was based on the hypothesised model (Figure 1) which was guided by existing literature on interprofessional collaboration [20-24,34,35], and pharmacist and GP interviews [15]. The preliminary version of the ATCI-P contained 21 items which covered ‘interactional’, ‘practitioner’ and ‘environmental determinants’. This version of the ATCI-P was distributed to 8 community pharmacists and 2 health services researchers with extensive experience in community pharmacy and general practice. This group was asked to comment on the face and content validity of the questionnaire, the appropriateness of the response options, whether items needed to be modified, removed or added as well as how the layout of the survey could be improved. Based on the feedback, modifications were made to the preliminary ATCI-P before it was pilot tested on 224 pharmacists in New South Wales, Australia. Principal Components Analysis (PCA) with oblimin rotation was then utilised to provide information regarding how each item contributed to the measurement of the construct. Items were evaluated for inclusion, exclusion or modification based on factor loadings, correlations and the interpretability of the item in relation to the extracted factor. Careful consideration of the PCA results led to a refined 15-item ATCI-P (Table 1).

**Table 1:** Survey items arranged by theme

**Interactional determinants**		
Communication	**ATCI-P 1**	The professional communication between myself and the GP is open and honest.
	**ATCI-P 8**	Discussions with the GP help me provide better patient care.
Trust	**ATCI-P 6**	I can trust the GP’s professional decisions.
	**ATCI-P 12**	I have confidence in the GP’s medical expertise.
Mutual respect	**ATCI-P 9**	The GP and I have mutual respect for one another on a professional level.
A willingness to work together	**ATCI-P 2**	The GP is open to working together with me on patients’ medication management.
	**ATCI-P 4**	The GP has time to discuss with me matters relating to patients’ medication regimens.
	**ATCI-P 10**	The GP and I share common goals and objectives when caring for the patient.
	**ATCI-P 15**	My working together with the GP benefits the patient.
**Environmental determinants**		
Proximity to GP’s office	*	In regards to the GP you have most dealings with, which of the following best describes the location of his/her surgery from your pharmacy?
System for collaboration	*	Do you have a system for working together with the GP with whom you have most dealings? For example, an agreed protocol for communication, a regular time for communication etc.?
Interprofessional education	*	Do you and the GP participate in joint continuing education events or meetings?
Remuneration	*	Does the availability of remuneration influence your decision to work with GPs in medication management?
Physician contact during training	*	During your pre-registration training did you have contact with GPs / Medical Officers regarding drug therapy?
**Practitioner determinants**		
Recognition of roles	**ATCI-P 11**	My role and the GP’s role in patient care are clear.
	**ATCI-P 13**	The GP believes that I have a role in assuring medication safety (for example, to identify drug interactions, adverse reactions, contraindications etc.)
	**ATCI-P 14**	The GP believes that I have a role in assuring medication effectiveness (for example, to ensure the patient receives the optimal drug at the optimal dose etc.)
Expectations	**ATCI-P 3**	The GP delivers high quality healthcare to patients.
	**ATCI-P 5**	The GP meets the professional expectations I have of him/her.
	**ATCI-P 7**	The GP actively addresses patients’ medical concerns.

For the refined ATCI-P, respondents were asked to indicate on a 5-point scale the extent to which they agreed or disagreed with a statement concerning themselves and the GP with whom they had most dealings. The points on the response scale were: 1 = ‘strongly disagree’, 2 = ‘disagree’, 3 = ‘neither agree nor disagree’, 4 = ‘agree’ and 5 = ‘strongly agree’.

### The questionnaire

The questionnaire consisted of the refined ATCI-P, a previously validated behavioural measure “Frequency of Interprofessional Collaboration Instrument for Pharmacists” (FICI-P) [36] and demographic questions. The FICI-P is a ten-item unidimensional measure that captures collaborative behaviour between pharmacists and GPs in primary care.

### Study sample

A sample of pharmacies stratified by state/territory was selected from a list of more than 4500 pharmacies in Australia. Australian postcodes were used to search online telephone directory White Pages® to generate a list which included all pharmacies in the database. One thousand two hundred and fifteen pharmacies were then selected and mailed a questionnaire addressed to ‘The Pharmacist’. The sample size (n ≥420) was calculated based on the number of responses required for Structural equation modelling (SEM) i.e. at least 10 responses per parameter estimate, then doubled to allow for a split sample validation analysis. Based on a response rate of 38% from a previous study [36], the minimum number of pharmacies that needed to be surveyed was calculated to be 420 / 0.38 = 1105.

### Data collection

The questionnaire was sent to the sample of 1215 pharmacies. The purpose and procedures of the study were described in an introductory letter included with the questionnaire and pharmacists were requested to return the survey in a reply-paid, self-addressed envelope. Questionnaires were marked with an ID number to allow for a follow-up/reminder mailing. Three weeks from the date the initial survey was distributed, all non-respondents were sent a reminder letter and another copy of the survey to complete. To encourage participation, respondents were entered into a draw for a chance to win one of four prizes: a double gold class movie pass, a bottle of champagne, a $100 shopping voucher or an iPod nano.

### Statistical analysis

Statistical analysis (PCA and SEM) was conducted using SPSS and AMOS 18.0. A preliminary analysis provided baseline descriptive statistics. Cases with missing data on variables of interest (FICI-P items, ATCI-P items and asterisked items presented in Table 1) were then removed and the sample was randomly divided into two groups. Sample 1 was used for initial testing of the structural model and Sample 2 was used for validation. The factor structure of the ATCI-P was investigated using PCA on Sample 1. The results from the PCA and the hypothesised theoretical model were used to guide the development of an empirically testable model to describe what influences interprofessional collaboration between GPs and pharmacists from the pharmacist’s perspective. The previously validated FICI-P [36] was used as a measure of pharmacist collaboration with GPs. The 10 items of the FICI-P were added to produce a score which represented pharmacists’ frequency of interprofessional collaboration with GPs and formed a basis for the construction of the model. The proposed model was then tested on Sample 2. The fit of the model was assessed using three indices:

a) Relative Chi-square (χ^2^/df): Used to determine the fit of the data to the model adjusted for the complexity of the model. Adequate model fit is obtained when χ^2^/df <3.

b) Root Mean-Square Error of Approximation (RMSEA) with 90% confidence level: Used to assess absolute fit of the model. Adequate model fit is obtained when RMSEA <0.08.

c) Bentler Comparative Fit Index (CFI): Used to assess incremental fit. Adequate model fit is obtained when CFI >0.90 [37].

Internal consistency of the two factors of the ATCI-P was assessed using Cronbach’s alpha.

## Results

Four hundred and ninety-two surveys were completed and returned for a response rate of 40%. Respondent characteristics are presented in Table 2. The gender and age distribution is similar to that reported in the 2006 Australian Census [38] which found 47% of retail pharmacists to be male and 39% to be aged 45 years or older. The distribution of pharmacists by state/territory is also similar to that reported in the Australian Government Department of Health and Ageing Annual Report 2005–2006 [39]. When cases with missing data (described above) were removed, 468 cases remained for analysis.

**Table 2:** Characteristics of pharmacist respondents (n = 492)

**Variable**	**Frequency (%)**
**Gender**	
Male	242 (49.2)
Female	250 (50.8)
**Age**	
<35 years	240 (48.8)
35–44 years	94 (19.1)
45–54 years	94 (19.1)
55–64 years	50 (10.2)
65 years +	8 (1.6)
Unspecified	6 (1.2)
**Years as a registered pharmacist**	
<20 years	310 (63.0)
20–39 years	164 (33.3)
40 years +	17 (3.5)
Unspecified	1 (0.2)
**Current position**	
Sole proprietor	116 (23.6)
Partner proprietor	92 (18.7)
Salaried manager	88 (17.9)
Pharmacist in charge	147 (29.9)
Locum pharmacist	10 (2.0)
Consultant pharmacist	4 (0.8)
Employee pharmacist	34 (6.9)
Unspecified	1 (0.2)
**Location of pharmacy by state / territory**	
Australian Capital Territory	9 (1.8)
New South Wales	151 (30.7)
Northern Territory	3 (0.6)
Queensland	99 (20.1)
South Australia	41 (8.3)
Tasmania	26 (5.3)
Victoria	105 (21.3)
Western Australia	58 (11.8)
**Location of pharmacy from GP with whom the pharmacist has most interactions**	
Co-located	38 (7.7)
Next door	79 (16.1)
Same shopping complex/strip	80 (16.3)
Less than 5 min walk away	171 (34.8)
More than 5 min walk away	122 (24.8)
Unspecified	2 (0.4)

PCA on Sample 1 (n = 234) produced a two factor solution (Table 3). ATCI-P items 1,2,4,8,9,10,11,13,14 and 15 made up Factor 1 and ATCI-P items 3,5,6,7 and 12 made up Factor 2. The items clustered together in a similar fashion as predicted (Table 1) with the exception of two themes: ‘recognition of roles’ (ATCI-P 11, 13 and 14) which loaded on ‘interactional determinants’ and ‘trust’ (ATCI-P 6 and 12) which loaded on ‘practitioner determinants’ in the PCA. As a result, Factor 1 was labelled as ‘interactional determinants’ and Factor 2 as ‘practitioner determinants’. Both factors showed good internal consistency with ‘interactional determinants’ and ‘practitioner determinants’ having a Cronbach’s α = .903 and .930 respectively (calculated using Sample 1 data).

**Table 3:** Factor structure of the ATCI-P (Sample 1 data)

**Item**		**Factor 1: Interactional determinants**	**Factor 2: Practitioner determinants**
ATCI-P 14	The GP believes that I have a role in assuring medication effectiveness (for example, to ensure the patient receives the optimal drug at the optimal dose etc.)	.938	
ATCI-P 13	The GP believes that I have a role in assuring medication safety (for example, to identify drug interactions, adverse reactions, contraindications etc.)	.883	
ATCI-P 15	My working together with the GP benefits the patient.	.747	
ATCI-P 9	The GP and I have mutual respect for one another on a professional level.	.632	
ATCI-P 8	Discussions with the GP help me provide better patient care.	.608	
ATCI-P 2	The GP is open to working together with me on patients’ medication management.	.597	
ATCI-P 11	My role and the GP’s role in patient care are clear.	.584	
ATCI-P 10	The GP and I share common goals and objectives when caring for the patient.	.575	
ATCI-P 1	The professional communication between myself and the GP is open and honest.	.531	
ATCI-P 4	The GP has time to discuss with me matters relating to patients’ medication regimens.	.439	
ATCI-P 6	I can trust the GP’s professional decisions.		-.901
ATCI-P 3	The GP delivers high quality health care to patients.		-.866
ATCI-P 5	The GP meets the professional expectations I have of him/her.		-.862
ATCI-P 12	I have confidence in the GP’s medical expertise.		-.853
ATCI-P 7	The GP actively addresses patients’ medical concerns.		-.802

Only loadings >.4 have been reported.

**Figure 2:**
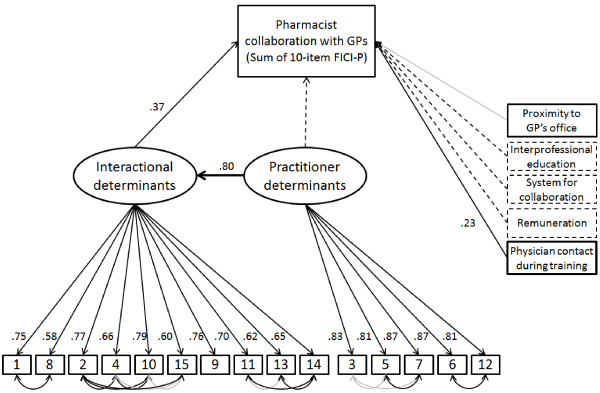
**Structural model showing factors influencing pharmacist collaboration with GPs.** For clarity, error terms have not been included in this figure. Dashed lines indicate relations that were modelled in the original structural model, but not in the modified structural model. Bold lines indicate relations added in the modified structural model. Values next to arrows are standardised coefficients. Coefficients displayed derive from data from the validation sample (Sample 2). Darker lines indicate significance at p <.001.

Results from the PCA were used to guide the construction of the structural model for interprofessional collaboration. Figure 2 shows the structural model with the individual items of the ATCI-P and factor loadings, combining the original structural model with the final modified structural model. The original structural model consisted of the sum of the 10-item FICI-P, the 15 items of the ATCI-P and 5 other observed variables (asterisked items in Table 1). The model fit of the original structural model (using Sample 1 data) was poor (χ^2^/df = 3.13, CFI = .82, RMSEA = .096, 90% CI [.087-.104]). The best model fit was obtained with the removal of 3 of the 5 observed variables, correlation of the ATCI-P items (based on theme, see Table 1) and the addition of a direct path from ‘practitioner determinants’ to ‘interactional determinants’ (rather than from ‘practitioner determinants’ to ‘Collaboration between pharmacist and GPs’). When the original structural model was modified this way, model fit improved (χ^2^/df = 1.89, CFI = .956, RMSEA = .062, 90% CI [.049-.074]). When this modified structural model was tested on Sample 2 (n = 234), model fit was also adequate (χ^2^/df = 1.89, CFI = .955, RMSEA = .062, 90% CI [.049-.074]). ‘Proximity to GP’s office’ and some of the modeled covariances, however, were not significant (Figure 2).

**Figure 3:**
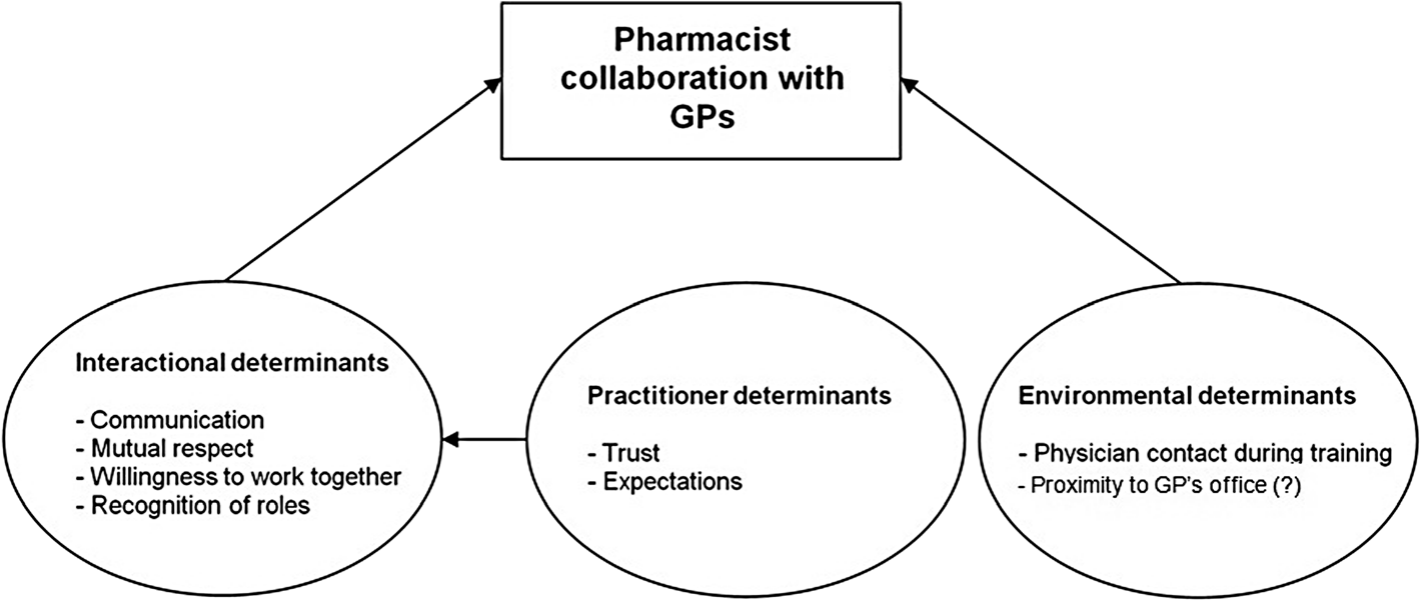
Validated model showing factors influencing pharmacist collaboration with GPs.

Figure 3 presents the modified structural model in a simplified format similar to the hypothesised model in Figure 1.

## Discussion

The results of the study provide evidence for the validity of the ATCI-P in measuring attitudes towards collaboration and illustrate the relationship between the frequency of interprofessional collaboration and ‘interactional’, ‘practitioner’ and ‘environmental determinants’. The structural model (Figure 2) describing these relations was modified and tested on a validation sample, and displayed adequate fit statistics. ‘Interactional determinants’ and an ‘environmental determinant’ were shown to influence collaboration directly, while ‘practitioner determinants’ was found to indirectly influence collaboration. ‘Interactional determinants’ was found to be the strongest predictor of pharmacist collaboration with GPs, and was in turn strongly influenced by ‘practitioner determinants’.

PCA of the ATCI-P indicated that items making up the variable ‘trust’ belong to ‘practitioner determinants’ rather than ‘interactional determinants’ as initially hypothesised (Figure 3). This may be because trust is linked to the individual rather than their interaction; that is, these items entail the pharmacist’s assessment of the GP, rather than an assessment of their interactions. It may be argued that a positive assessment of a practitioner is in fact a prerequisite for strong interactions. In contrast ‘recognition of roles’ was found to belong to ‘interactional determinants’ rather than ‘practitioner determinants’. This may be because perceptions on role affect how practitioners interact with one another.

Regarding the ‘environmental determinants’, only ‘physician contact during training’ was found to be a predictor of collaboration. The item asks pharmacists whether during their pre-registration training they rarely, occasionally or frequently had contact with GPs (if training was carried out in community pharmacy) or medical officers (if training was carried out in hospital pharmacy). Those pharmacists who had frequent contact with GPs and/or medical officers during their pre-registration training were more likely to have higher levels of collaboration with their GP counterpart in their current practice. This may suggest that exposure to collaboration during the final year of pharmacist training equips pharmacists with the skills and confidence for future collaboration.

When ‘proximity to GP’s office’ was modelled as a predictor of collaboration using Sample 1 data, those pharmacists working in closer proximity to their GP counterparts were found to have higher levels of collaboration than isolated practitioners. This may be because being geographically closer to one another provides more opportunity to develop rapport and positive relationships as a result of increased interaction. However when ‘proximity to GP’s office’ was modelled and tested on the validation sample (Sample 2), the impact of this variable was not replicated. As this variable has been identified as important in previous studies [15,27], and significantly impacted on collaboration when modelled using Sample 1 data, it should not be disregarded but may be worth investigating in future research.

This study has several important implications for practice and highlights possible strategies for improving interprofessional collaboration between pharmacists and GPs. Policy makers may wish to consider strategies for fostering good communication, trust and respect between GPs and pharmacists. One strategy may be to restructure primary health care services so that GPs and pharmacists are collocated. This would make them more accessible to one another and thereby increase opportunities for interprofessional collaboration. For pharmacist collaboration with GPs to be successful there must also be a willingness from both parties to work together. This may be nurtured by creating educational opportunities that allow pharmacists more interaction with their medical colleagues in their formative years to build confidence and encourage teamwork. It would also allow GPs to recognise and appreciate that pharmacists have an important contribution to make to medication safety and effectiveness.

Several limitations to the study should be noted: firstly, the response rate of 40% was only modest however the size of the sample was adequate for the analysis. Secondly, the items that make up the instruments are reflective of current primary care practice and may require refinement if changes to practice occur in the future. This, however, is not a shortcoming of the theoretical model proposed, but rather a qualification that the specific items employed in the model must align with current practice. It should also be noted that the ATCI-P and model have been developed for community pharmacists practicing in Australia. Validity testing of the ATCI-P and model for other settings e.g. ambulatory and tertiary settings; for other practitioners e.g. GPs or hospital pharmacists; and in other countries may yield different results and requires further research. Finally, as pharmacists have different interactions with different GPs it was necessary to ask the respondents to think of only one GP when completing the questionnaire. Respondents were asked to ‘think of the GP with whom you have most dealings’. Therefore, the results may be biased towards reflecting the relationship of more actively collaborating pharmacist and GP pairs than that of the average population.

## Conclusions

The results of the study provide evidence for the validity of the ATCI-P in measuring attitudes towards collaboration and support a model of collaboration in which collaborative behaviour is influenced directly by ‘interactional’ and ‘environmental determinants’, and indirectly by ‘practitioner determinants’. The proposed model provides valuable insight and a good foundation for policy makers, researchers and practitioners to develop strategies for pharmacists to improve collaboration with GPs in the interest of quality use of medicines and improved health outcomes. The FICI-P and ATCI-P may also be useful tools for determining the extent of collaboration in current practice and will allow for empirical assessment of interventions targeted at enhancing pharmacist collaboration with GPs.

## Appendix

### Frequency of Interprofessional Collaboration Instrument for Pharmacists (FICI-P)

1. The GP and I openly communicated with each other.

2. I informed the GP of new products/services that are available/that I provide.

3. The GP contacted me for specific drug information.

4. The GP contacted me for specific patient information.

5. I contacted the GP to clarify scripts.

6. I contacted the GP to discuss dosage adjustments.

7. I contacted the GP to recommend an alternative medication (e.g. due to an adverse reaction, contraindication etc).

8. The GP adjusted patient medication after my recommendation.

9. The GP shared patient information with me.

10. The GP involved me in decisions regarding medication management.

### Attitudes Towards Collaboration Instrument for Pharmacists (ATCI-P)

1. The professional communication between myself and the GP is open and honest.

2. The GP is open to working together with me on patients’ medication management.

3. The GP delivers high quality healthcare to patients.

4. The GP has time to discuss with me matters relating to patients’ medication regimens.

5. The GP meets the professional expectations I have of him/her.

6. I can trust the GP’s professional decisions.

7. The GP actively addresses patients’ medical concerns.

8. Discussions with the GP help me provide better patient care.

9. The GP and I have mutual respect for one another on a professional level.

10. The GP and I share common goals and objectives when caring for the patient.

11. My role and the GP’s role in patient care are clear.

12. I have confidence in the GP’s medical expertise.

13. The GP believes that I have a role in assuring medication safety (for example, to identify drug interactions, adverse reactions, contraindications etc).

14. The GP believes that I have a role in assuring medication effectiveness (for example, to ensure the patient receives the optimal drug at the optimal dose etc).

15. My working together with the GP benefits the patient.

**Note:** In answering both the FICI-P and ATCI-P items, respondents were asked to ‘think of a GP with whom you have most dealings’. In answering the FICI-P items, respondents were asked to ‘estimate the number of times the following has occurred in the last month’ on a 4-point response scale, where 1 = ‘nil’, 2 = ‘1-2 times’, 3 = ‘3-4 times’ and 4 = ‘5 or more times.’ In answering the ATCI-P items, respondents were asked to ‘indicate the extent to which you agree or disagree with the following statements’ on a 5-point response scale, where 1 = ‘strongly disagree’, 2 = ‘disagree’, 3 = ‘neither agree nor disagree’, 4 = ‘agree’ and 5 = ‘strongly agree’.

## Competing interests

The authors declare that they have no competing interests.

## Authors’ contributions

CV contributed to the development of the questionnaire, performed the statistical analysis and drafted the manuscript. DC contributed to the development of the questionnaire, performed the statistical analysis and revised the manuscript. PA contributed to the development of the questionnaire and revised the manuscript. BM contributed to the development of the questionnaire and revised the manuscript. IK conceived of the study, contributed to the development of the questionnaire and revised the manuscript. All authors read and approved the final manuscript.

## Pre-publication history

The pre-publication history for this paper can be accessed here:

http://www.biomedcentral.com/1472-6963/12/320/prepub
